# Editorial: Causes and Consequences of Sleep Apnea: Spotlights on the Roles of Sex and Sex Hormones

**DOI:** 10.3389/fphys.2022.857627

**Published:** 2022-02-23

**Authors:** Vincent Joseph, Silvia Pagliardini, Elise Belaidi

**Affiliations:** ^1^Département de Pédiatrie, Faculté de Médecine, Centre de Recherche de l'Institut Universitaire de Cardiologie et Pneumologie de Québec, Université Laval, Quebec, QC, Canada; ^2^Department of Physiology, Faculty of Medicine and Dentistry, University of Alberta, Edmonton, AB, Canada; ^3^Women and Children Health Research Institute, University of Alberta, Edmonton, AB, Canada; ^4^Université Grenoble Alpes, INSERM, CHU Grenoble Alpes, HP2, Grenoble, France

**Keywords:** sleep apnea (SA), sex, sex hormones, cardiovascular diseases, sympathetic nervous system, metabolic dysfunction

Sleep apnea (SA) is a highly prevalent respiratory disease associated with cardiovascular, neurological, or metabolic pathologies (Eisele et al., 2015; Gileles-Hillel et al., 2016). For decades, SA has been considered a men's disease due to the large male prevalence reported in early studies (Block et al., 1979). However, it is now largely acknowledged that in adults roughly 1/3 of all SA patients are women, and that following menopause the prevalence increases in women to reach levels similar than in men (Heinzer et al., 2015; Basoglu and Tasbakan, 2018; Sunwoo et al., 2018). More concerning is the fact that women are less likely to be diagnosed than men due to the misconception that women are at very low risk, but also to the reported difference in symptoms and signs of the disease (Mallampalli and Carter, 2014; Basoglu and Tasbakan, 2018). There are moreover evidences that women are underrepresented in SA clinics and research protocols (Redline et al., 1994), although recent studies are contributing to reduce this gap (Mokhlesi et al., 2016; Basoglu and Tasbakan, 2018; Heinzer et al., 2018). The aims of this Research Topic were therefore to highlight (1) recent studies addressing the differences in comorbidities between men and women, and between pre- and post-menopausal women, (2) the different end-organ dysfunctions and cellular alterations as reported in SA animal models, and (3) the different mechanisms by which sex hormones can interact with intermittent hypoxia to alter the cellular responses and modify the co-morbidities of SA in men and women.

Martins and Conde have produced a detailed review presenting the links between obstructive SA (OSA) and metabolic dysfunctions with a focus on sex and sex-hormones. Basic concepts and relevant data linking OSA and dysmetabolism through hormonal mediators (insulin, leptin, ghrelin, and orexin pathways, HPA axis) or through inflammation and oxidative stress are presented. Importantly, this paper thoroughly review the evidence gathered from animal and clinical studies supporting the hypothesis that carotid bodies (CB) contribute to metabolic dysfunction in SA patients. Indeed, intermittent hypoxia (the major OSA consequence) enhances sympathetic nervous system (SNS) activity through CB peripheral chemoreceptors, and elevated SNS activity is a major driver of metabolic alterations. Moreover, sex-hormone receptors are present in CB and other central nuclei that regulate SNS activity providing the basis to hypothesize a probable mechanism underlying a sex-specific metabolic consequences of SA. The review by Hegner et al. present recent data showing that women with SA are more likely to develop heart failure with preserved ejection fraction compared to men, with a positive correlation between SA severity and echocardiographic alteration (diastolic left ventricular filling). On the other hand, heart failure with reduced ejection fraction is more frequent in men than in women. In this review, the authors also discussed sex-specific differences of endothelial dysfunction, arterial hypertension, stroke, and atrial fibrillation, with presentation of putative interactions with systemic inflammation, oxidative stress, and Ca^2+^ homeostasis in cardiomyocytes putting forwards to establish new testable hypothesis for future animal or clinical studies.

Next are two original articles that further expand the Research Topic by covering specific populations in which incidence of OSA is expected to be elevated, namely pregnant women (Panyarath et al.), and a cohort of healthy elderly subjects (>65 yrs—Vacelet et al.). In the first study, Panyarath et al. sought to address whether hypertension disorder of pregnancy (HDP) is associated with Obstructive Sleep Apnea Hypopnea (OSAH) syndrom. While there was no correlation between the Apnea Hypopnea Index (AHI) and mean 24-h blood pressure, almost 80% of the women with OSAH and HDP in this study were not showing the expected nocturnal decrease of SBP, which is a sign of altered SNS regulation. Furthermore, there were significant inverse relationships between AHI and the % decrease of nocturnal mean, systolic and diastolic blood pressures in these women. This is an important and intriguing study, clearly supporting usefulness of OSAH treatment strategies to mitigate obstetrical risks in pregnant women.

The study of Vacelet et al. assessed potential associations between OSA and diabetes mellitus along with insulin resistance in a community-based cohort. Subjects (66.2 ± 1 years old) were followed for 7 years to assess risk of diabetes (defined as fasting glucose ≥ 1.26 g/L or requiring treatment). Their results indicated that their risk was not elevated in asymptomatic and untreated OSA subjects and was not associated with sleep fragmentation or hypoxic stress in men and women alike. On the other hand, the risk of insulin resistance (HOMA-IR ≥ 2) was 2.2-fold higher in severe OSA patients, although multivariate analysis indicated that this was most likely linked to obesity status, fasting glucose, and triglycerides levels rather than hypoxic loads. These are intriguing results that contribute to a better understanding of the metabolic consequences of OSA in this specific population (i.e., OSA asymptomatic patients ≥ 65 years).

Animal research is an unvaluable tool to assess how sex and sex hormones determine the origin or consequences of SA in males and females (Ribon-Demars et al., 2018; Laouafa et al., 2019; Marcouiller et al., 2021; Tenorio-Lopes and Kinkead, 2021). The paper by Elliot-Portal et al. uses neonatal maternal separation (NMS) to induce a stress response in mice pups and examine the long-term consequences. In adulthood, NMS mice exhibit more SA and higher hypercapnic ventilatory response than controls. Notably, this was only present in males, and the breathing defects induced by NMS in males was prevented by overexpression of erythropoietin (EPO) in the brain of transgenic mice. This is a striking example that a multitude of factors shape respiratory behavior in adult mammals, some of them likely contributing to the SA occurrence. Because EPO is a promising neuroprotective agent in preterm neonates (Wang et al., 2020), its proposed long-term respiratory effects should not be overlooked, specifically in male neonates. These findings might provide a useful model to better understand why OSA is more prevalent in infants and young adults that are born before term (Gauda and McLemore, 2020).

## Author Contributions

VJ wrote the editorial. EB and SP commented and edited the text. All authors approved the final version.

## Conflict of Interest

The authors declare that the research was conducted in the absence of any commercial or financial relationships that could be construed as a potential conflict of interest.

## Publisher's Note

All claims expressed in this article are solely those of the authors and do not necessarily represent those of their affiliated organizations, or those of the publisher, the editors and the reviewers. Any product that may be evaluated in this article, or claim that may be made by its manufacturer, is not guaranteed or endorsed by the publisher.
